# Evaluation of the Relationship between Left Coronary Artery Bifurcation Angle and Coronary Artery Disease: A Systematic Review

**DOI:** 10.3390/jcm11175143

**Published:** 2022-08-31

**Authors:** Jade Geerlings-Batt, Zhonghua Sun

**Affiliations:** Discipline of Medical Radiation Science, Curtin Medical School, Curtin University, Perth, WA 6845, Australia

**Keywords:** coronary computed tomography angiography, coronary artery disease, coronary artery bifurcation, atherosclerosis, coronary stenosis

## Abstract

Recent studies have suggested a relationship between wide left coronary artery bifurcation (left anterior descending [LAD]-left circumflex [LCx]) angle and coronary artery disease (CAD). Current literature is multifaceted. Different studies have analysed this relationship using computational fluid dynamics, by considering CAD risk factors, and from simple causal-comparative and correlational perspectives. Hence, the purpose of this systematic review was to critically evaluate the current literature and determine whether there is sufficient evidence available to prove the relationship between LAD-LCx angle and CAD. Five electronic databases (ProQuest, Scopus, PubMed, CINAHL Plus with Full Text, and Emcare) were used to locate relevant texts, which were then screened according to predefined eligibility criteria. Thirteen eligible articles were selected for review. Current evidence suggests individuals with a wide LAD-LCx angle experience altered haemodynamics at the bifurcation site compared to those with narrower angles, which likely facilitates a predisposition to developing CAD. However, further research is required to determine causality regarding relationships between LAD-LCx angle and CAD risk factors. Insufficient valid evidence exists to support associations between LAD-LCx angle and degree of coronary stenosis, and future haemodynamic analyses should explore more accurate coronary artery modelling, as well as CAD progression in already stenosed bifurcations.

## 1. Introduction

Coronary artery disease (CAD) is the leading cause of morbidity and mortality in developed countries, comprising 11 percent of deaths in Australia in 2018 [1], as well as over 87 percent of the total national health expenditure [2], with expenditure referring to money spent on health goods and services, which does not include burden of disease-related costs and losses in productivity [1]. CAD involves the development of atherosclerotic plaques within the coronary arteries, resulting in coronary artery stenosis [2,3], and progressive deposition of these plaques entails increasingly compromised vessel patency, which often precedes acute coronary syndrome (ACS) [2,3]. ACS occurs due to impaired coronary circulation and encompasses a range of debilitating and often life-limiting conditions, such as unstable angina and myocardial infarction with and without ST segment elevation [3]. These individual and collective impacts emphasise the importance of understanding CAD pathogenesis in order to improve patient outcomes and mitigate the associated burden of disease.

**Figure 1:**
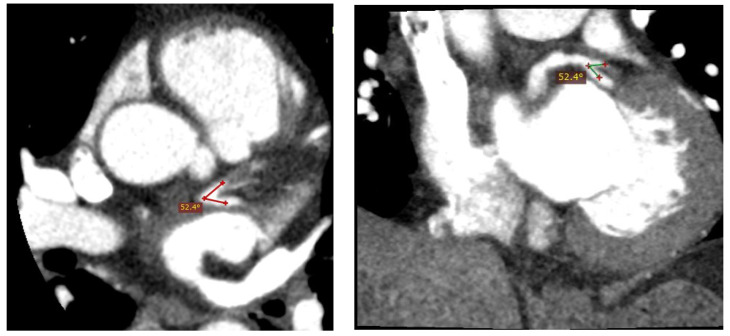
Narrow LAD-LCx angle in a 50-year male patient with no coronary artery disease. The LAD-LCx angle was measured 52.4° on 2D axial image (**left** image) and multiplanar reformatted view (**right** image).

**Figure 2:**
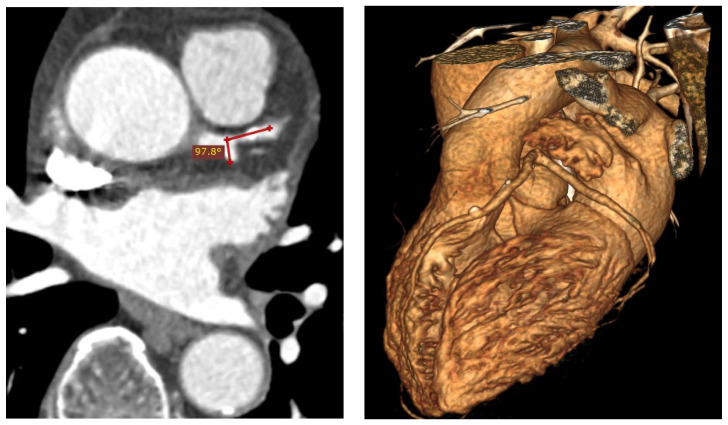
Wide LAD-LCx angle in a 68-year-old man with multiple calcified plaques at the LAD resulting in significant stenosis. The LAD-LCx angle was measured 97.8° on 2D axial image (**left** image) and 103.5° on 3D volume rendering (**right** image), respectively.

One of the most common coronary sites for atherosclerotic plaque formation is the proximal left anterior descending (LAD) artery, particularly at the bifurcation site where the LAD and left circumflex (LCx) arteries meet [4,5], as shown in Figure 1 and Figure 2. The findings of several recent studies have suggested a direct correlation between LAD-LCx bifurcation angle and CAD [4,6,7,8]. This has been investigated further via computational fluid dynamics (CFD), with the correlation being primarily attributed to a change in wall shear stress (WSS) observed in bifurcation angles of variable size [9,10,11,12]. Consequently, it has been proposed that the LAD-LCx angle may be indicative of a patient’s likelihood of developing CAD, and it could be a useful tool for enhancing the routine diagnostic reporting of coronary computed tomography angiography (CCTA) studies [4,13], especially since the LAD-LCx measurement is of improved diagnostic value compared to standard coronary lumen assessment [6]. 

The relationship between LAD-LCx angle and CAD has been analysed from multiple perspectives, and has been investigated using CFD, coronary computed tomography angiography (CCTA), as well as via a retrospective review of patient demographic data. Additionally, the strength and direction of the relationships between LAD-LCx angle, and CAD and associated risk factors are described differently amongst current literature. Hence, readers of this scattered, multifaceted discussion may find it difficult to decipher. The purpose of this review is to critically evaluate the current literature and investigate the relationship between LAD-LCx angle and CAD with regard to relationship direction, fluid dynamics, and CAD risk factors. This review aims to address and identify areas requiring further exploration, and assess the discrepancies, strengths, and limitations of current evidence to guide future studies investigating the relationship between LAD-LCx angle and CAD. 

## 2. Materials and Methods

This review was guided by the research question, “Is there sufficient evidence to prove the relationship between LAD-LCx angle and if so, should measurement of LAD-LCx angle be incorporated into the routine diagnostic reporting of CCTA?”—and a search strategy was subsequently devised. Literature was filtered according to predefined inclusion and exclusion criteria, and the key characteristics and findings of the included publications were consolidated in an evidence table, in order to aid in effective synthesis and critical appraisal of the literature. 

### 2.1. Search Strategy

A systematic literature search was conducted using the online databases ProQuest, Scopus, PUBMED (including Medline), Cumulative Index to Nursing and Allied Health (CINAHL) Plus with Full Text, and Emcare. The utilised search strategy is summarised in Table 1.

The review was performed to comply with the preferred reporting items for systematic reviews and meta-analyses (PRISMA) guidelines and the search was limited to peer-reviewed articles published in English between 2005 and 2022. This publication date range was selected in an attempt to achieve some semblance of currency, as the technology used to investigate the relationship between LAD-LCx angle and CAD, such as CFD software and CCTA, has likely improved significantly since pre-2005. CAD can be referred to in a number of ways and to avoid inadvertently excluding relevant publications, several iterations of CAD, (“coronary artery disease” OR “coronary heart disease” OR stenosis OR atherosclero*), were included as search terms, and “left main coronary” OR “left coronary” was included to capture literature discussing the left coronary vessels, specifically. Similarly, multiple phrases and truncations were defined, (“coronary bifurcat*” OR “bifurcat* angl*” OR “artery bifurcat*” OR angul* OR angle*), in order to capture articles specifically relating to the coronary bifurcation angle. The search initially included a large number of articles investigating the mechanism of restenosis post left coronary stenting, which were irrelevant for the purpose of this review, and were consequently excluded by including NOT stent* in the search. 

### 2.2. Eligibility Criteria

Full text, original, peer-reviewed, empirical research papers exploring the relationship between LAD-LCx angle and CAD were included in this review. Articles written in a non-English language were excluded, as were publications inaccessible as a full text and articles published prior to 2005. Duplicate papers were removed, and the titles and abstracts of the remaining articles were screened for relevance. Since this review aimed to evaluate the naturally occurring relationship between LAD-LCx angle and CAD, any articles investigating post-interventional phenomena were also excluded. Articles with titles explicitly referencing post-interventional stenosis were excluded. Full texts were subsequently assessed for eligibility. Articles not specifically discussing LAD-LCx angle, and/or its relationship with CAD were irrelevant for the purpose of this review and were excluded.

### 2.3. Data Extraction and Analysis

The following details were extracted from the eligible studies: authors and year of publication, sample size, study design and methodology in terms of whether assessing the LAD-LCx angle in relation to CAD or using computational flow dynamic analysis to determine haemodynamic changes at the left coronary bifurcation region, and key findings, as well as study strengths and limitations. A modified Cochrane risk of bias assessment tool was used to assess the included causal-comparative studies for detection, missing data, reporting, and other types of bias [14]. Reference searching and data extraction was mainly performed by one assessor (J.B) with results validated by another assessor (Z.S).

## 3. Results

**Figure 3:**
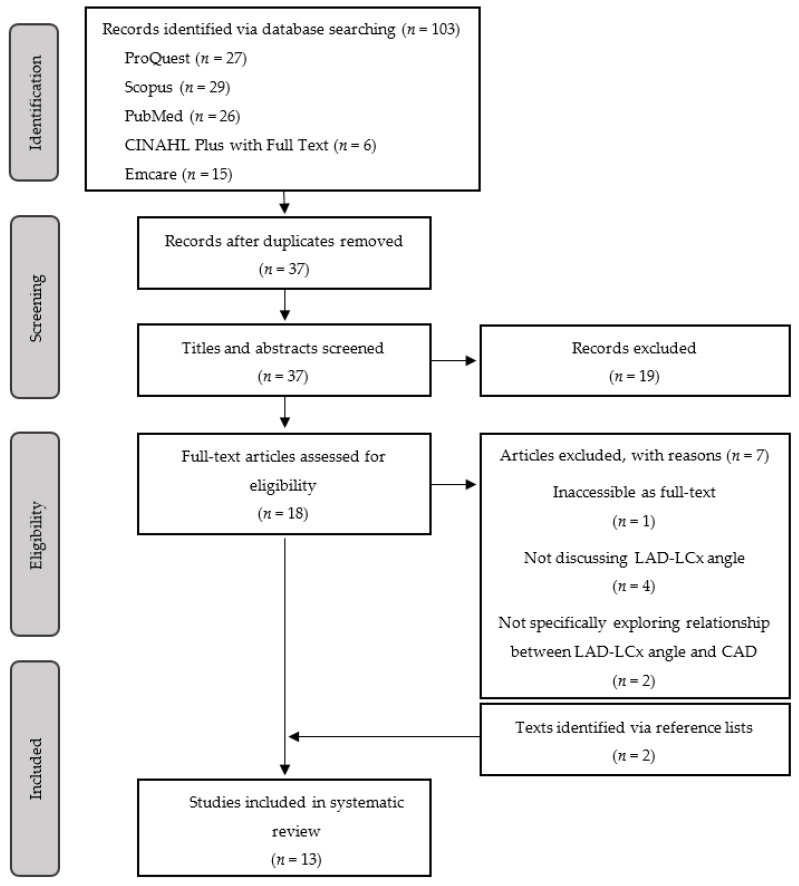
PRISMA flow diagram to search for relevant studies.

The search initially returned a total of 103 records across the five selected databases. Sixty-six duplicates were excluded, and the titles of the remaining 37 records were screened for eligibility. Several articles with titles describing irrelevant subject matter were excluded, such as those referencing interventional techniques for treating coronary bifurcation stenosis. One article was unavailable as a full text and was not included in this review. Of the remaining 18 full texts, four articles did not analyse LAD-LCx angle, and two did not specifically assess the relationship between LAD-LCx angle and CAD, and were consequently also excluded. The reference lists of the included texts were examined for texts with eligible titles, and two additional articles were identified for inclusion. Thirteen articles were found to meet the specified eligibility criteria and were included in this review (Figure 3). 

### Study Characteristics

Seven of the thirteen studies were causal-comparative by design [4,6,7,8,15,16,17], five were descriptive [9,10,11,12,18], and one study had both causal-comparative and correlational components [19] (Table 2). Five of the thirteen studies (38%) were conducted in Australia [4,6,10,11,16], three (22%) in China [8,9,12], one (8%) in Taiwan [7], one (8%) in Turkey [19], one (8%) in South Korea [15], one (8%) in Argentina [17], and one (8%) was a collaborative study between authors from Saudi Arabia, India, and Oman [18]. Five studies (38%) involved the use of CFD analysis to observe and measure intraluminal forces at the LAD-LCx angle [9,10,11,12,18], and eight (62%) involved repeatedly measuring LAD-LCx angle on coronary angiography imaging to assess its relationship with CAD and/or associated risk factors [4,6,7,8,15,16,17,19]. Of the studies analysing CFD, one used models derived from real patients’ CCTA datasets [10], two used simulated LAD-LCx models [12,18], and two used a combination of both [9,11], with sample sizes ranging from four to 30 models [9,10,18]. Of the eight studies using coronary angiography to assess the relationship between LAD-LCx and CAD, seven used CCTA [4,6,7,8,15,16,17], and one used invasive coronary angiography (ICA) to measure LAD-LCx angle [9], with sample sizes ranging from 30 [6] to 467 patients [19]. Two of these studies also investigated possible relationships between various CAD risk factors and LAD-LCx angle [4,7]. Three of the eight studies subdivided their CAD group according to degree of coronary stenosis, with two of these using ICA [7,8] and one using CCTA to determine the degree of coronary stenosis [15]. LAD-LCx angle cut-off values for predicting coronary stenosis were calculated in five of the included studies [7,8,10,15,19], and values ranged from 60° [15] to 80.5° [19].

Table 2 summarises study design, key findings, strengths, and limitations. The findings from these 13 studies were summarised in five areas with regard to the relationship between LAD-LCx angle/haemodynamic changes and CAD development which are considered in Section 4. Table 3 outlines the risk of detection, missing data, and reporting bias for the eight included causal-comparative studies according to the Cochrane risk of bias assessment tool [14]. The remaining five studies were not included in this risk of bias assessment since the utilised tool was inapplicable to their descriptive methodologies.

## 4. Discussion

Upon analysis of the included literature, several themes emerged. Current literature has discussed relationships between LAD-LCx angle and CAD, degree of stenosis, and other variables such as plaque type and location, and analysed intraluminal forces using CFD. Limitations of the current literature largely surround an inability to determine causality, and a restricted volume of valid evidence relating to certain relationships.

### 4.1. LAD-LCx Angle and CAD

**Figure 4:**
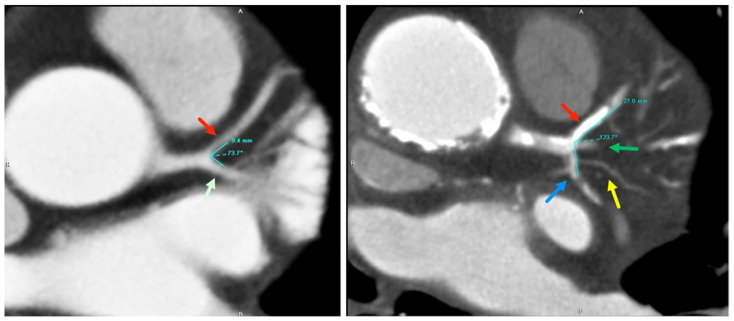
Measurement of LAD-LCx angles on 2D axial images in both normal and diseased cases. Left image: The LAD-LCx angle was measured as 73.7° in a 53-year-old man with normal findings at LAD, while in another patient, 73-year-old male with significant stenosis of the LAD, the angle was measured as 123.7°. Reprinted with permission under the open access from Juan et al. [7] 2017, Public Library of Science.

The current literature provides evidence to suggest that wide LAD-LCx angle is associated with CAD [6,7,8,17]. Between the eight causal-comparative studies investigating this relationship, both CCTA and ICA were used to measure LAD-LCx angle. Whilst ICA has traditionally been considered the gold standard for coronary artery evaluation, both modalities are valid tools for measuring coronary angles [13]. CCTA has proven to be an accurate tool for assessing LAD-LCx angle (Figure 4), exhibiting comparable, if not superior sensitivity, specificity, and positive predictive values to those of standard lumen assessment by ICA [13]. 

Sun [16] calculated an LAD-LCx angle of 80° as a significant cut-off value for predicting CAD, which has been supported by several studies reporting similar values [7,8,13]. However, it is unclear whether this threshold value is simply predicting the presence of CAD, or rather luminal narrowing greater than a certain degree within a specified vessel. For example, whilst there is a claimed consensus between studies, a cut-off value was calculated by Sun [16] to predict left coronary stenosis, by Cui et al. [8] to predict left coronary stenosis >50%, by Juan et al. [7] to predict CAD, and by Moon et al. [15] to predict LAD stenosis >50%. Additionally, the value calculated by Moon et al. deviated greatly at 60°, which was attributed to an unspecified bias associated with the study’s small sample size. Hence, accurate interpretation of this information is difficult, due to the incongruity existing between studies. 

**Figure 5:**
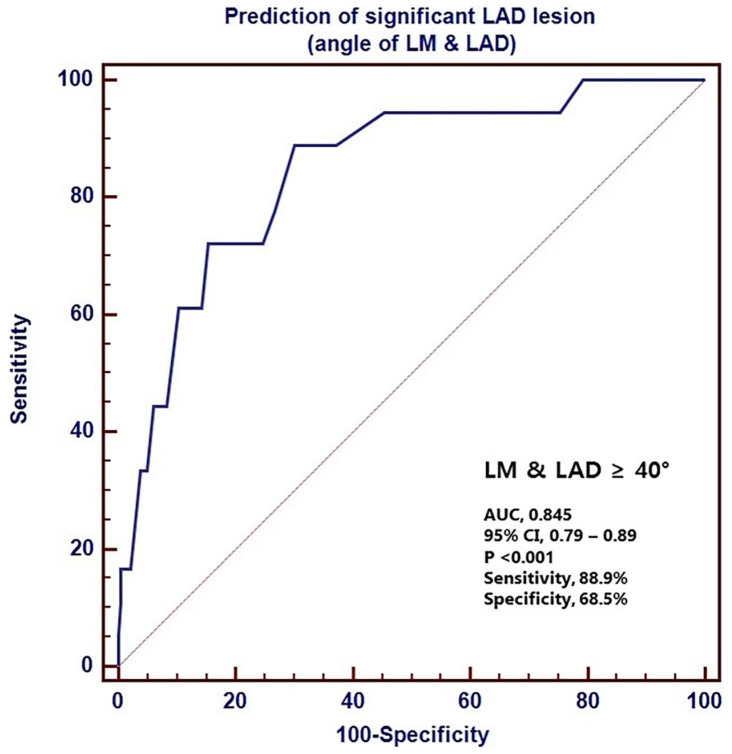
The receiver operating characteristic (ROC) curve for the LM-LAD angle in the prediction of LAD stenosis. The area under the curve is 0.845. LM—left main coronary artery, LAD—left anterior descending, CI—confidence interval. Reprinted with permission under the open access from Moon et al. [15] 2018, Public Library of Science.

**Figure 6:**
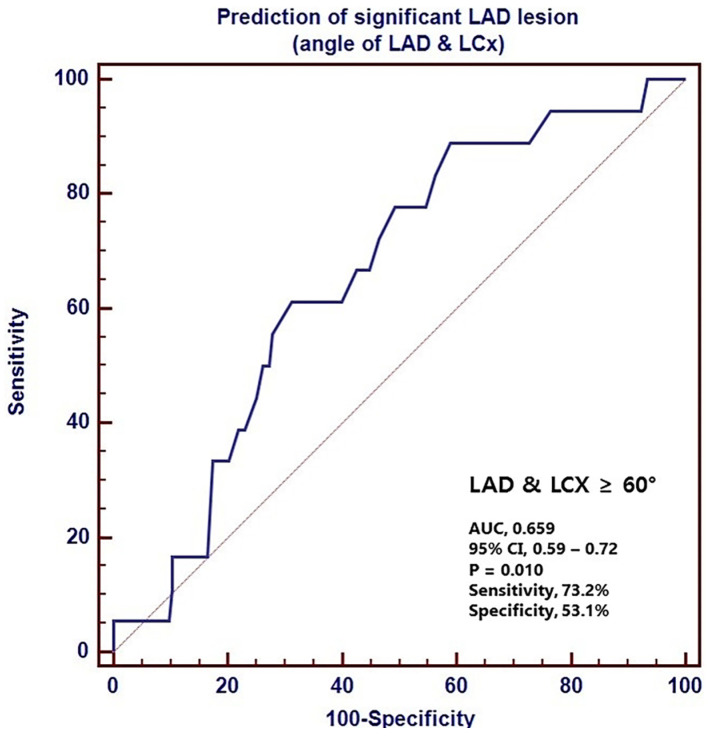
The receiver operating characteristic (ROC) curve for the LAD-LCx angle in the prediction of LAD stenosis. The area under the curve is 0.659. LAD—left anterior descending, LCx—left circumflex, CI—confidence interval. Reprinted with permission under the open access from Moon et al. [15] 2018, Public Library of Science.

Moon et al. [15] found the LM-LAD angle to be a significant predictor for LAD stenosis >50%, postulating that WSS within the LAD may be affected by wide LM-LAD angle, as opposed to wide LAD-LCx angle. Hence, CAD within the LAD, specifically, may not be correlated with wide LAD-LCx angle, but instead with LM-LAD angle. This cannot be determined from the current literature, given that four of the five included CFD studies increased the LAD-LCx angle by reducing both the LM-LAD and LM-LCx angles [9,11,12,18] which may be confounding. Liu et al. [9] also suggested that wide LM-LAD may be related to the development of CAD, however, LM-LAD angle was defined and measured differently by Moon et al. [15] and consequently, the findings of these two studies are contradictory (Figure 5 and Figure 6). Further research is necessary to develop these theories and establish whether variable LM-LAD and LM-LCx angle size is associated with stenosis in specific vessels. 

### 4.2. LAD-LCx Angle and Other Variables

There is minimal evidence available supporting relationships between LAD-LCx angle and other variables, such as CAD risk factors, plaque type. and location. Temov et al. [4] investigated the relationships between LAD-LCx angle and sex, BMI, hypertension, cholesterol, diabetes, smoking, and family history, as predefined CAD risk factors. The findings of this study suggested males and individuals with a BMI > 25 kg/m^2^ were more likely to exhibit an LAD-LCx angle > 80°, compared to females and individuals with a BMI < 25 kg/m^2^, respectively [4]. The relationship between LAD-LCx angle and sex was also supported by two subsequent studies [7,19] and Juan et al. [7] attributed larger LAD-LCx angles among males to their generally larger body habitus, theorising consequently expanded coronary angles. BMI may influence coronary bifurcation angles in a similar manner. Epicardial adipose tissue covers approximately 80% of the cardiac surface, insulating the myocardium and existing between the coronary vessels. Analysis of histological and imaging data has indicated that relative epicardial fat mass increases with BMI [20]. Whilst the increased epicardial fat volume associated with obesity may play a role in widening coronary angles, there is no evidence available to support or challenge this theory. Wide LAD-LCx angle may also be correlated with atherosclerotic lesions closer to the LCA bifurcation site [19], as well as non-calcified plaques [8]. However, literature discussing relationships between LAD-LCx angle and other variables related to CAD is scarce. If correlations between LAD-LCx angle and these other variables are to be more thoroughly understood, further research is required in this area. 

### 4.3. LAD-LCx Angle and Degree of Coronary Stenosis

Current literature suggests that LAD-LCx angle is significantly wider among individuals with coronary stenosis >50%, compared to those with <50% coronary stenosis [8,15]. Whilst Juan et al. [7] did not observe a relationship between LAD-LCx angle and degree of stenosis, this may be due to reduced study validity; a portion of their CCTA datasets was low quality, and one of their subsamples consisted of significantly fewer patients compared to the other two groups. Although CCTA is a valid tool for measuring LAD-LCx angle [7,13] the appearance of severe atherosclerotic calcification on CCTA is exacerbated by blooming artefacts caused by partial volume averaging [21,22,23]. This reduces the specificity and overall validity of CCTA as a tool for assessing degree of coronary stenosis. ICA is instead considered the gold standard for identifying obstructive coronary lesions and measuring luminal narrowing in cases of extensive coronary calcification [13]. However, Moon et al. [15] measured luminal narrowing solely on CCTA, and the authors do not disclose whether individuals without CAD were included in the group with <50% stenosis. Despite being consistent in their findings, respective errors in the methods of these two studies may have restricted the validity of their results, and some uncertainty remains regarding the relationship between LAD-LCx angle and the degree of coronary stenosis.

### 4.4. Computational Fluid Dynamics

**Figure 7:**
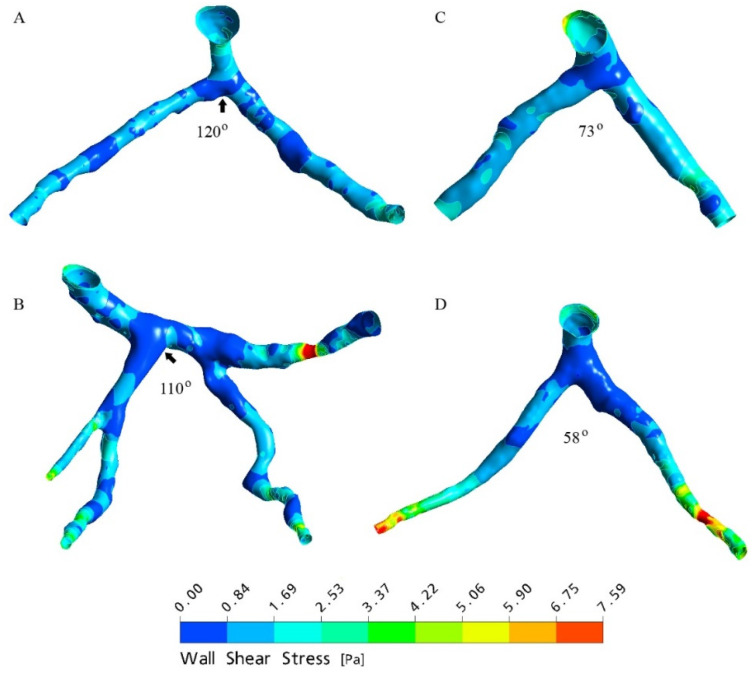
Wall shear stress observed with variable angles of the realistic left coronary artery models generated at peak systolic phase of 0.4 s. (**A**,**B**) refer to models with wide LAD-LCx angles, while (**C**,**D**) refer to models with narrow LAD-LCx angles. Arrows refer to the low wall shear stress distributions at large bifurcations. Reprinted with permission from Chaichana et al. [11] 2011, Elsevier.

**Figure 8:**
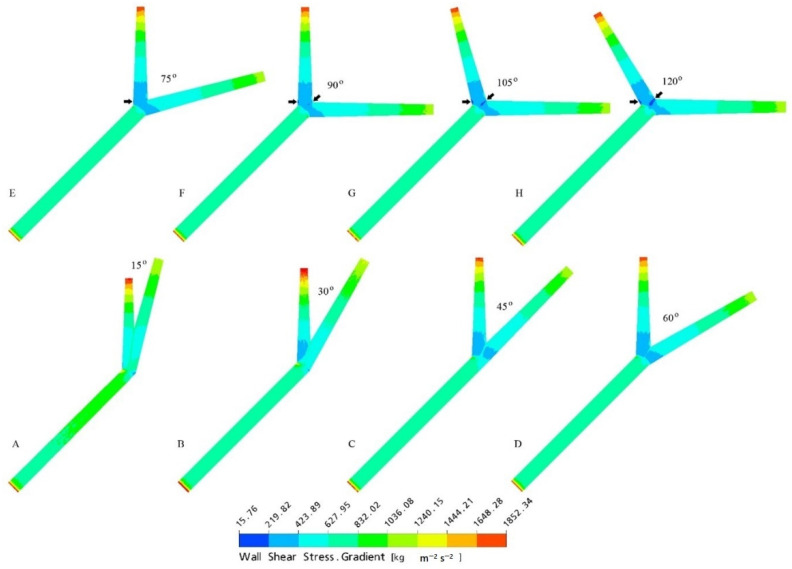
Wall shear stress gradient observed with variable angles of the simulated left coronary artery models generated at peak systolic phase of 0.4 s. (**A**–**H**) simulated coronary artery models with different LAD-LCx angles ranging from narrow to wide angulation. Arrows refer to the wall shear stress gradient distributions and a big region of the low magnitude present at 120° angulation model. Reprinted with permission from Chaichana et al. [11] 2011, Elsevier.

Multiple CFD studies have correlated reduced WSS at the LCA bifurcation site with wide LAD-LCx angle [9,10,11,12], supporting the theory of WSS playing a pivotal role in preventing the development of CAD (Figure 7). Low WSS has been observed to correspond to low flow velocity [11], and wide LAD-LCx angles were also associated with increased wall pressure in the LAD and LCx arteries in LCA bifurcations unaffected by CAD [10,11]. Wide bifurcations also exhibited larger regions of low WSS compared to those observed in narrow bifurcations [12]. Kamangar et al. [18] used CFD to study the haemodynamics of the LCA bifurcation with an 80% stenosed LAD artery. The relationship between LAD-LCx angle and WSS was the inverse of that observed in simulated bifurcations without luminal narrowing, high WSS, and flow velocity and was noted at the area of stenosis in models with wide LAD-LCx angle [18]. Similarly, Sun and Chaichana [10] observed significantly reduced wall pressure at stenotic regions, and increased flow velocity at post stenotic regions within the LAD and LCx arteries in models with wide LAD-LCx angle. This is unusual considering that wide LAD-LCx angles are associated with more severe coronary stenosis, despite the increased flow velocity and WSS observed in stenotic CFD modelling. Further research is required to properly understand the relationship between haemodynamics and CAD progression in already stenosed coronary bifurcations. Although several studies propose WSS to be a primary determining factor for developing atherosclerosis, Chaichana et al. [11] hypothesised wall shear stress gradient (WSSG) to be a more reliable indicator of at-risk regions within the LCA bifurcation (Figure 8). However, subsequent studies are yet to develop this theory by analysing the distribution of WSSG. 

Although some CFD studies included only simulated models [12,18] whilst others included models derived from patients’ CCTA datasets [9,10,11], their findings did not differ greatly, and the strengths and limitations of the included CFD studies are otherwise consistent. Simulations consistently assumed a rigid (rather than an elastic) arterial wall [9,10,11,12], limiting the extent to which coronary physiology was represented. This was predicted to have had negligible effect on the validity of the simulations, especially in studies analysing stenotic vessels exhibiting restricted wall elasticity [18]. CFD simulations typically used Newtonian fluid models for their simplicity [9,11,12], which is inaccurate considering the non-Newtonian properties of blood and its related fluid-structure interactions [9,11]. Whilst Kamangar et al. [18] used a non-Newtonian fluid model, their findings are of limited generalisability given the study analysed only the haemodynamics of an LCA bifurcation affected by severe LAD stenosis. Although these limitations were assumed to have minimal effect on study validity, future research should incorporate non-Newtonian fluid models and elastic wall modelling to achieve more accurate simulation of coronary physiology. Use of machine learning or deep learning for rapid and accurate CFD analysis of haemodynamics in the coronary arteries could be a research direction as already shown in many studies investigating the CT-derived fractional flow reserve [24,25,26,27].

### 4.5. Causality

The current evidence supporting the relationship between wide LAD-LCx angle and CAD consists predominantly of causal-comparative findings. Causal-comparative and correlational studies are useful for discovering relationships between dependent and independent variables where true experimental research is impossible or unethical, but these studies are inherently limited by their inability to determine causality [28,29]. This is also true of descriptive studies, where observers can establish whether relationships exist, but cannot accurately determine cause and effect [28]. Several studies have established that there is a relationship between wide LAD-LCx angle and CAD [6,7,8,16], but it is impossible to determine the direction of causality from these studies alone; whether the development of CAD is influenced by LAD-LCx angle, or whether changing LAD-LCx angle is a consequence of CAD. However, CFD analysis of non-stenosed vessels has given plausibility to the theory that wide LAD-LCx angle increases a person’s likelihood of developing CAD, as opposed to LAD-LCx angle being influenced by the development of CAD. If causality is to be properly established between LAD-LCx angle and other previously discussed variables, subsequent investigators could consider using a framework, such as the Bradford Hill Criteria [30,31] to properly evaluate the likelihood of a causal relationship. 

### 4.6. Current Limitations and Recommendations for Future Research

A knowledge gap exists in the current literature regarding its ability to determine causality, and the evidence provided supporting a relationship between LAD-LCx angle and CAD is limited due to the reduced validity of the included studies. WSSG, LM-LAD, and LM-LCx angles, and the haemodynamics of already stenosed LCA bifurcations also require further exploration to reinforce current evidence. Research regarding LAD-LCx angle and CAD is yet to correlate angle measurement by CCTA with patient outcomes. Hence, future research should incorporate prospective study designs with patient follow-up to ascertain the value of using LAD-LCx measurement in routine diagnostic CCTA reporting. Due to limited diagnostic value of CCTA in assessing calcified plaques, use of high-resolution CT or deep learning approaches to improve the specificity and positive predictive value of CCTA in heavily calcified plaques could further elucidate the correlation between left coronary angulation and CAD [32,33,34,35,36,37].

## 5. Conclusions

There is a relationship between wide LAD-LCx angle and CAD, which is likely due to the reduced WSS observed in wide LCA bifurcations. Further haemodynamic analyses are required to explore non-Newtonian fluid and elastic arterial wall modelling, and the effects variable LM-LAD and LM-LCx angles on haemodynamics, as well as to better understand the pathogenesis CAD progression in already stenosed LCA bifurcations. Several studies have investigated correlation, but due to the inherent limitations of causal-comparative study designs, they have been unable to properly determine the possibility of causality for various relationships, such as for the observed correlations between LAD-LCx angle, and sex, BMI, plaque type, and lesion distance from the LCA bifurcation site. Future research should endeavour to further characterise these associations and investigate causal relationships. 

## Figures and Tables

**Table 1:** Search strategy with use of a combination of key words.

Boolean Operator	Term	Field
	“coronary artery disease” OR “coronary heart disease” OR stenosis OR atherosclero*	Title
AND	“left main coronary” OR “left coronary”	Title/abstract
AND	“coronary bifurcat*” OR “bifurcat* angl*” OR “artery bifurcat*” OR angul* OR angle*	Title
NOT	stent*	Title

**Table 2:** Study characteristics, strengths, limitations, and findings.

Reference	Country of Origin	Study Aim	Sample Size	Study Design	Strengths	Limitations	Key Findings
Chaichana et al. [11] (2011)	Australia	To investigate the haemodynamic effect of variable LAD-LCx angulation using realistic and simulated LAD-LCx models.	12 models	Descriptive	Realistic modelling derived from CCTA datasets of real patients Wall shear stress gradient (WSSG) analysed Relatively large sample size for CFD study Analysed several different angle sizes	Simulations assumed a rigid arterial wall Incorporated a Newtonian fluid model Did not account for pathological changes	Regions of low wall shear stress (WSS) corresponded to regions of low flow velocity Reduced wall pressure, WSS and flow velocity was observed at the bifurcation sites of all models Models with wide LAD-LCx angles demonstrated reduced WSS and WSSG at the bifurcation site compared to those with narrow angles Wall pressure in the LAD and LCx arteries was higher in models with a wider LAD-LCx angle, than those with a narrow angle
Cui et al. [8] (2017)	China	To evaluate the value of LAD-LCx angles and plaque characteristics as predictors of coronary stenosis by dual-source computed tomography.	106 patients	Causal-comparative	LAD-LCx angles were measured on CCTA Degree of coronary stenosis was determined by ICA Explored relationships between LAD-LCx angle and degree of coronary stenosis	Single-centre study Authors state “more participants are required to verify [their] results,” but do not discuss the extent to which their study was limited by their sample size Did not explore relationships between CAD risk factors and LAD-LCx angle, despite collecting this demographical data	Wide LAD-LCx angle was associated with significant left coronary stenosis and non-calcified plaques LAD-LCx angle was significantly wider in patients with ≥50% left coronary stenosis, than those with <50% left coronary stenosis LAD-LCx angle of 78° was calculated as a cut-off value for predicting significant left coronary stenosis
Juan et al. [7] (2017)	Taiwan	To understand the relationship between LAD-LCx angle and CAD in patients with normal, non-significantly and significantly stenosed left coronary arteries.	313 patients	Causal-comparative	LAD-LCx angles were measured on CCTA Explored relationships between LAD-LCx angle and several CAD risk factors Cross tabulated LAD-LCx with multiple variables via logistic regression Relatively large overall sample size Degree of coronary stenosis was determined by ICA	Study validity may have been affected by the small size of group III compared to groups I and II A proportion of the CCTA datasets were of lower quality than the remainder of the sample, which may have affected angle measurements	LAD-LCx angle was significantly wider in patients with ≥50% coronary stenosis than those with normal coronary arteries LAD-LCx angle of 80° was calculated as a cut-off value for predicting left coronary stenosis Males and patients with high body mass index (BMI) were significantly more likely to have a LAD-LCx angle > 80°, compared to females and patients with low BMI, respectively
Kamangar et al. [18] (2020)	Saudi Arabia, India and Oman	To compare the effect of bifurcation angle on haemodynamic parameters in the left coronary artery with 80% stenosis.	4 models	Descriptive	Analysed the haemodynamics of stenotic bifurcations Incorporated a non-Newtonian fluid model	Sample consisted of simulated models only Simulations assumed a rigid arterial wall Only investigated haemodynamics of 80% stenosed vessels Very few angles were studied	Wall pressure at the stenotic region was significantly reduced with wide LAD-LCx angle, compared to narrow LAD-LCx angle Flow velocity at the stenotic region increased with LAD-LCx angle WSS at the area of stenosis in models with wide LAD-LCx angle was higher than in those with narrow angles High WSS was observed at the stenotic region in all models
Liu et al. [9] (2019)	China	To investigate the effect of different bifurcation angles on the left coronary artery.	4 models	Descriptive	Used one realistic model derived from the CCTA dataset of a real patient	Simulations assumed a rigid arterial wall Incorporated a Newtonian fluid model Very few angles were studied	Models with wide LAD-LCx angles demonstrated reduced WSS at the bifurcation site compared to those with narrow angles The angle between the left main coronary artery and the LAD artery (LM-LAD angle) influences WSS Models with a narrow left main-LAD (LM-LAD) angle demonstrated greater WSS at the bifurcation site than those with wide LM-LAD angle
Moon et al. [15] (2018)	South Korea	To evaluate the associations between the left main-LAD and LAD-LCx angles, and LAD stenosis.	201 patients	Causal-comparative	LAD-LCx angles were measured on CCTA Relatively large sample size	Unclear whether group with ≤50% stenosis included patients without CAD Degree of coronary stenosis was measured on CCTA Calculated LAD-LCx cut-off value for predicting CAD lower than several other studies	LAD-LCx angle was significantly wider amongst patients with ≥50% stenosis, compared to those with ≤50% stenosis LAD-LCx angle of 60° was calculated as a cut-off value for predicting left coronary stenosis LM-LAD angle was significantly wider amongst patients with CAD, compared to those without CAD
Rodriguez-Granillo et al. [17] (2007)	Argentina	To explore plaque burden at different segments of the left main bifurcation and its relationship with bifurcation angle using high-resolution multislice CT coronary angiography.	50 patients	Causal-comparative	LAD-LCx angles were measured on CCTA	Relatively small sample size Degree of coronary stenosis was not considered	Wide LAD-LCx angle was closely related to the presence of plaques within the left coronary artery bifurcation
Sun [16] (2013)	Australia	To investigate the relationship between intraluminal appearances of coronary plaques and left coronary bifurcation angle and plaque components using coronary CT virtual intravascular endoscopy (VIE).	50 patients	Causal-comparative	LAD-LCx angles were measured on CCTA Explored possible relationships between LAD-LCx angle and intraluminal appearances	Degree of coronary stenosis was not considered CCTA datasets were acquired on three different CT machines with three different imaging protocols Relatively small sample size	LAD-LCx angle was significantly wider amongst patients with left CAD, compared to those with a normal left coronary artery The mean diameters of LAD and LCx in patients with left coronary disease and a LAD-LCx angle > 80° were significantly larger than those with left coronary disease and a LAD-LCx angle < 80°
Sun & Cao [6] (2011)	Australia	To investigate the relationship between left coronary bifurcation and dimensional changes and development of CAD using CT angiography.	30 patients	Causal-comparative	LAD-LCx angles were measured on CCTA Findings are supported by several other studies Valid statistical tests were conducted	Degree of coronary stenosis was not considered Relatively small sample size	LAD-LCx angle was significantly wider amongst patients with left CAD, compared to those without left CAD 89% of patients with both LAD and LCx disease had a bifurcation angle > 90%
Sun & Chaichana [10] (2017)	Australia	To investigate the correlation between LAD-LCx angle and coronary stenosis, as assessed via CCTA-generated CFD analysis.	30 models	Descriptive	Realistic modelling derived from CCTA datasets of real patients Degree of coronary stenosis was determined by ICA Relatively large sample size for a CFD study	Simulations assumed a rigid arterial wall Incorporated a Newtonian fluid model Authors do not provide their definition for ‘significant coronary stenosis’ Number of cases with significant coronary stenosis was small Study focused on calcified plaques	Increased WSS was observed in the LAD and LCx arteries of models with significant coronary stenosis and a LAD-LCx angle > 80° Wall pressure decreased at stenotic regions in patients with wide LAD-LCx angles Flow velocity increased at post-stenotic regions in the LAD and LCx arteries with significant stenosis LAD-LCx angle of 80° was calculated as a cut-off value for predicting significant left coronary stenosis
Temov & Sun [4] (2016)	Australia	To explore the association between LAD-LCx angle and common atherosclerotic risk factors with regard to CAD development using CCTA.	196 patients	Causal-comparative	LAD-LCx angles were measured on CCTA Relatively large sample size Explored relationships between LAD-LCx angle and several CAD risk factors	Degree of coronary stenosis was not considered Multivariate analysis was not conducted	Males were significantly more likely to have a LAD-LCx angle > 80°, compared to females Patients with a BMI > 25 kg/m^2^ were significantly more likely to have a LAD-LCx angle > 80°, compared to those with a BMI < 25 kg/m^2^
Zhang et al. [12] (2016)	China	To determine whether there is a relationship between bifurcated arterial geometry and haemodynamics.	7 models	Descriptive	Wall pressure gradient (WPG) was analysed in addition to WSS	The sample consisted of simulated models only Simulations assumed a rigid arterial wall Incorporated a Newtonian fluid model	Models with wide LAD-LCx angle had larger low WSS regions, compared to those with narrow LAD-LCx angle Models with wide LAD-LCx angle demonstrated smaller regions of low WPG at the bifurcation site, compared to models with narrow LAD-LCx angle
Ziyrek et al. [19] (2020)	Turkey	To analyse the effect of coronary bifurcation angle and left main coronary artery length on atherosclerotic lesion localisation.	467 patients	Causal-comparative Correlational	LAD-LCx angles were measured on ICA Relatively large sample size Degree of coronary stenosis was determined by ICA Performed correlational analysis	Number of patients with significant ≥50% coronary stenosis was small Focused only on plaques located in close proximity to the LCA bifurcation site	LAD-LCx angle of 80.5° was calculated as a cut-off value for predicting atherosclerotic lesion/s located ≤5 mm from the bifurcation site Wide LAD-LCx angle was strongly correlated with lesions located closer to the LCA bifurcation site LAD-LCx angle was significantly wider amongst males, compared to females

BMI—body mass index, CAD—coronary artery disease, CCTA—coronary computed tomography angiography, CFD—computational fluid dynamics, ICA—invasive coronary angiography, LAD—left anterior descending, LCA-left coronary artery, LCx—left circumflex, WSS—wall shear stress, WSG—wall pressure gradient.

**Table 3:** Risk of bias assessment among the studies that were reviewed.

Reference	Bias Domain	Risk Level	Support for Judgement
Cui et al. [8] (2017)	Selection bias *	-	-
	Performance bias ^#^	-	-
	Detection bias	Unclear	Authors do not disclose whether measurements were conducted by a blind assessor.
	Missing data bias ^^^	Unclear	Authors do not specify whether cases were omitted due to incomplete data.
	Reporting bias	Low	All prespecified outcomes were reported.
Juan et al. [7] (2017)	Selection bias *	-	-
	Performance bias ^#^	-	-
	Detection bias	High	Assessors measuring coronary angles were not blinded.
	Missing data bias	Unclear	Authors do not specify whether cases were omitted due to incomplete data.
	Reporting bias	Low	All prespecified outcomes were reported.
	Other: Measurement bias	Unclear	An unspecified portion of the acquired CCTA datasets from each group were of relatively low spatial resolution, which may have affected the accuracy of subsequent measurements for those cases.
Moon et al. [15] (2018)	Selection bias *	-	-
	Performance bias ^#^	-	-
	Detection bias	Unclear	Authors do not disclose whether measurements were conducted by a blind assessor.
	Missing data bias ^^^	Unclear	Authors do not specify whether cases were omitted due to incomplete data.
	Reporting bias	Low	All prespecified outcomes were reported.
	Other: Measurement bias	Unclear	Degree of coronary stenosis was measured solely on CCTA, and its appearance may have consistently been exacerbated in cases from the group with ≥50% stenosis due to blooming artefact associated with extensive calcification.
Rodriguez-Granillo et al. [17] (2007)	Selection bias *	-	-
	Performance bias ^#^	-	-
	Detection bias	Unclear	Authors do not disclose whether measurements were conducted by a blind assessor.
	Missing data bias ^^^	Unclear	Authors do not specify whether cases were omitted due to incomplete data.
	Reporting bias	Low	All prespecified outcomes were reported.
Sun [16] (2013)	Selection bias *	-	-
	Performance bias ^#^	-	-
	Detection bias	Unclear	Authors do not disclose whether measurements were conducted by a blind assessor.
	Missing data bias ^^^	Unclear	Authors do not specify whether cases were omitted due to incomplete data.
	Reporting bias	Low	All prespecified outcomes were reported.
Sun & Cao [6] (2011)	Selection bias *	-	-
	Performance bias ^#^	-	-
	Detection bias	Unclear	Authors do not disclose whether measurements were conducted by a blind assessor.
	Missing data bias ^^^	Unclear	Authors do not specify whether cases were omitted due to incomplete data.
	Reporting bias	Low	All prespecified outcomes were reported.
Temov & Sun [4] (2016)	Selection bias *	-	-
	Performance bias ^#^	-	-
	Detection bias	Unclear	Authors do not disclose whether measurements were conducted by a blind assessor.
	Missing data bias ^^^	High	An unspecified number of cases with unavailable CAD risk factor checklists were omitted from the study; coronary angle measurements were not completed for these cases.
	Reporting bias	Low	All prespecified outcomes were reported.
Ziyrek et al. [19] (2020)	Selection bias *	-	-
	Performance bias ^#^	-	-
	Detection bias	Unclear	Authors do not disclose whether measurements were conducted by a blind assessor.
	Missing data bias ^^^	Unclear	Authors do not specify whether cases were omitted due to incomplete data.
	Reporting bias	Low	All prespecified outcomes were reported.

*: Due to the causal-comparative nature of these studies, degree of selection bias associated with inadequate randomization cannot be properly assessed, since these are not randomized controlled trials. ^#^: Performance bias cannot be assessed due to the exclusively retrospective nature of the included studies. ^^^: Missing data bias was discussed instead of attrition bias for all studies, also due to their retrospective, causal-comparative designs.

## Data Availability

Not applicable.
